# Knowledge, Attitudes and Practices Regarding Livestock Diseases Among Residents of East Gojjam Zone, Amhara Region, Ethiopia

**DOI:** 10.1002/vms3.70743

**Published:** 2025-12-28

**Authors:** Liuel Yizengaw, Haregua Yesigat, Kifile Wondimagegnehu, Arega Tafere, Habtamu Yalew, Dessalew Habtie, Natenael Teshager, Yibeltal Simeneh

**Affiliations:** ^1^ Department of Veterinary Laboratory Technology College of Agriculture and Natural Resource Debre Markos University Debre Markos Ethiopia; ^2^ School of Veterinary Medicine Wollo University Dessie Ethiopia; ^3^ School of Veterinary Medicine Bahir Dar University Bahir Dar Ethiopia; ^4^ East Gojjam Zone Livestock and Fishery Developmental Office Debre Markos Ethiopia

**Keywords:** attitude, disease, East Gojjam, knowledge, livestock, practice

## Abstract

The prevention and management of livestock diseases require a holistic approach. Among these, the knowledge, attitudes and practices (KAP) of livestock owners play a central role. KAP surveys provide valuable insights into how individuals perceive livestock health challenges, what preventive measures they implement and how they respond to disease outbreaks. This study, therefore, aims to assess the KAP related to livestock diseases and their determinants among the residents of East Gojjam Zone, Amhara Region, to provide evidence‐based insights for policymakers, extension workers and other stakeholders working to improve animal health in the region. A cross‐sectional study design was employed based on a questionnaire survey of 412 participants from five selected districts of the East Gojjam Zone, Amhara Region. A multistage cluster sampling procedure was used to select participants. Quantitative score was generated for KAP, and the scores were dichotomized as adequate and inadequate knowledge, desirable and undesirable attitude and good and poor practice. Descriptive statistics and multiple logistic regression were used to see the association of predictor variables towards adequate knowledge, desirable attitude and good practice. About 57%, 69% and 49% of the respondents have adequate knowledge, desirable attitude and good practice scores, respectively. About 96% of participants explained that feed and free grazing land was the livestock production constraint followed by disease (92%). Majority of the participants (83%) agree that livestock is an important economic and zoonotic disease that can threaten the lives of humans and animals. Only 32% and 21% of respondents used personal protective equipment during the sick animal approach and disposed of their dead animal through burial and burning. Illiterate level participants were less likely to have adequate knowledge than college and above level (AOR = 0.24, 95% CI = 0.06–0.87), and in the same way, respondents of illiterate individuals were less likely to have good practice than college and above level participants (AOR = 0.04, CI = 0.009–0.19). The study showed that participants have good knowledge and attitude towards livestock disease but poor disease prevention and control practices. The community public health education should focus on translation of these good knowledge and favourable attitude into practices that effectively reduces livestock disease burden of the community.

## Graphical abstract

This study reveals gaps in livestock owners’ knowledge and practices regarding disease prevention and control in East Gojjam Zone, Ethiopia, highlighting the need for strengthened veterinary extension services and community education to improve animal health management.

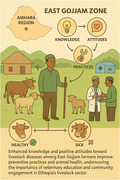

## 1 Background

Livestock production has been considered as one of the main components of agricultural development in most parts of sub‐Saharan Africa. Livestock production is the main source of livelihoods and nutrition for over 300 million people residing in sub‐Saharan Africa (Abdilatif et al. 2018). Livestock farming remains a critical component of Ethiopia's agricultural economy, contributing about 40% of agricultural GDP and supporting the livelihoods of over 65 million people (Brief 2004; Mekuriaw and Harris‐Coble 2021). In the East Gojjam Zone of the Amhara Region, rural communities largely depend on mixed crop–livestock production systems, where animals such as cattle, sheep, goats, poultry and equines are vital not only for food security and income but also for draught power, manure and sociocultural functions (Berihu and Tamir 2015).

The estimated livestock population of Ethiopia is 71.1 million heads of cattle, 42.8 million heads of sheep, 52.3 million heads of goats, 57.1 million chickens, 14.4 million equine and 6.96 million beehives (Begna and Masho 2024). From these animal populations, Amhara Region has cattle (14,710,911), sheep (10,024,277), goat (6,064,944), horses (420,760), mules (157,213), donkeys (2,677,429), camels (66,364), poultry (18,031,121) and beehives (1,361,329; Begna and Masho 2024; Mekuriaw and Harris‐Coble 2021; Leta and Mesele 2014). Like other area of the region, East Gojjam Zone is one having a large population of livestock, which has the estimated livestock population of cattle (2,407,345), sheep (951,982), goat (549,778), horses (31,096), mules (29,088), donkey (258,407), poultry (1,924,685) and beehives (241,713; Worku et al. 2024).

Despite its socioeconomic importance, the livestock sector is persistently challenged by a high burden of diseases. Endemic and transboundary diseases such as foot‐and‐mouth disease (FMD), anthrax, brucellosis, lumpy skin disease (LSD) and various internal and external parasites contribute to reduced productivity, increased mortality, poor market performance and escalating treatment costs (Alemu et al. 2021; Tenagne et al. 2023). In addition to economic losses, zoonotic diseases such as brucellosis and anthrax pose significant risks to public health, especially in communities with close human–animal interaction (WHO 2020).

The prevention and management of livestock diseases require a holistic, one health–based approach involving animal, human and environmental health sectors (WHO 2024), incorporating access to veterinary services, routine vaccination, good husbandry practices and informed community participation (Moges and Bogale 2012). Among these, the knowledge, attitudes and practices (KAP) of livestock owners play a central role. When farmers are well‐informed about disease signs, transmission routes and preventive measures, they are more likely to adopt timely and effective interventions. Conversely, a lack of awareness or misconceptions about disease causes and treatments can lead to delayed responses, increased spread and poor health outcomes for both animals and humans (Alders et al. 2020). Studies in other parts of Ethiopia and sub‐Saharan Africa have shown that farmer awareness and perceptions critically influence their decisions regarding disease reporting, treatment seeking and preventive actions (Tufa et al. 2023; Mukarugwiro 2021; Jibat et al. 2015; Dernburg et al. 2007).

The knowlege, attitude and practices (KAP) framework has been widely used in public health and veterinary research to identify behavioural patterns and inform community‐based interventions. KAP surveys provide valuable insights into how individuals perceive livestock health challenges, what preventive measures they implement and how they respond to disease outbreaks (Govindaraj et al. 2016). Understanding the community's KAP regarding livestock diseases is essential for designing appropriate interventions, improving animal health and enhancing livelihoods (Jost et al. 2010; Moges and Bogale 2012). However, empirical data on the KAP of livestock owners in the East Gojjam Zone remain limited, hindering the design of context‐specific veterinary extension programmes.

Existing extension efforts are often constrained by logistical barriers, low literacy levels and cultural beliefs that affect information dissemination and uptake (Amghani et al. 2025; Nettle et al. 2022; Anugwa 2018). This underscores the need for localized, evidence‐based interventions that consider the socioeconomic and educational context of farmers in East Gojjam. The objective of this study was to assess the KAP of livestock owners in East Gojjam Zone regarding common livestock diseases, to provide evidence‐based insights for policymakers, extension workers and other stakeholders working to improve animal health in the region.

## 2 Materials and Methods

### 2.1 Study Area and Study Population

**FIGURE 1:**
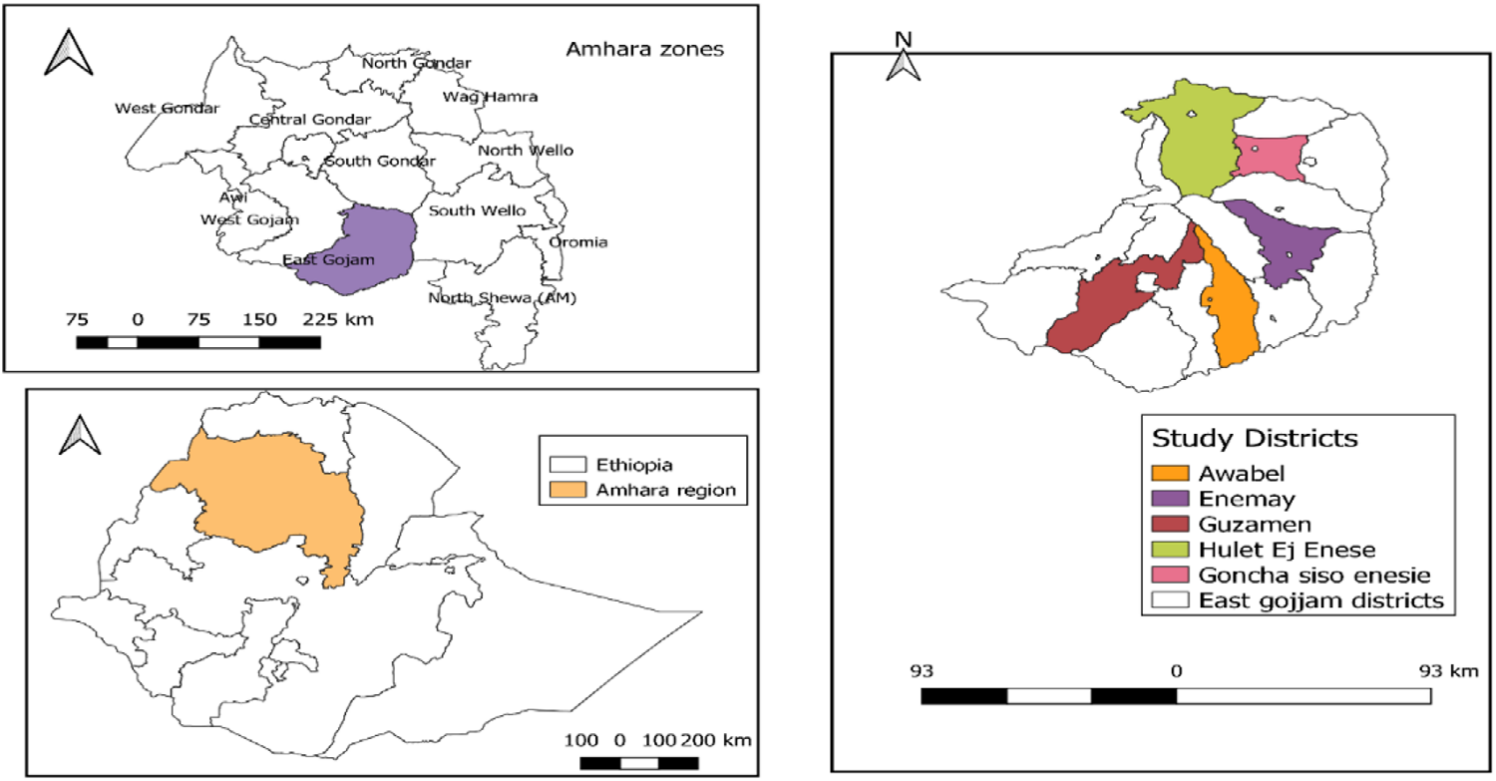
Map of the study area showing the Amhara Region within Ethiopia, study zone within the Amhara Region and districts with in East Gojjam Zone.

This study was conducted in the East Gojjam Zone, which is one of the administrative zones of the Amhara Regional State in northwestern Ethiopia. The zone is geographically located between 10°00′ and 11°30′ N latitude and 37°30′ and 38°45′ E longitude. It comprises 20 woredas (districts), including both rural and urban areas the estimated total population of 2,351,855 and 506,520 households with an estimated area of 14,004.47 km^2^; this zone has an estimated density of 170 people per square km (Carbonetti et al. 2024). Debre Markos is the administrative centre of the zone and serves as a key commercial and service hub. The area is characterized by diverse agroecological zones highland, midland and lowland, supporting a mixed crop–livestock farming system, with a significant portion of the population depending on livestock for income, food and draft power. Five districts, Awabel, Enemay, Gozamen, Goncha Siso Enesie and Hulet Eju Enesie, were included in the study (Figure 1). Livestock production plays a vital role in the livelihoods of residents in the East Gojjam Zone, with cattle, sheep, goats, equines and poultry being the predominant species reared. The study population consisted of households involved in livestock farming residing in selected kebeles (the smallest administrative units) within the East Gojjam Zone. Participants were adult household members primarily responsible for livestock management. Inclusion criteria were residence in the area for more than 6 months and willingness to participate in the study. Local veterinary professionals and development agents were also consulted to provide supplementary insights into disease patterns and community practices.

### 2.2 Study Design and Data Collection

The cross‐sectional study design was used to assess the KAP of livestock owners regarding livestock diseases in East Gojjam Zone, Amhara Region, Ethiopia, in which selected individuals from the community were interviewed once during April to December 2024 using a structured questionnaire. The questionnaire was designed to collect quantitative data on participants’ knowledge, attitude and practice related to livestock diseases (ruminant, shoat and equine) and variables that could affect knowledge, attitude and practice. The questionnaire contains questions about knowledge, attitude and practice and potential sociodemographic and animal management factors that could affect them. The questionnaire was developed based on comprehensive livestock disease‐related literature reviews and discussions among researchers. A structured questionnaire contains a total of 54 questions, which were categorized in to five sections: socioeconomic characteristics (10), animal demography and management practice (6), knowledge (15), attitude (11) and practice (12) of respondents related to livestock disease. In the knowledge and practice question, respondents were asked to answer ‘yes’ or ‘no’ or to choose from a list of options provided. Attitude questions were developed in a bipolar Likert's scale having five components: *strongly disagree*, *disagree*, *uncertain*, *agree and strongly agree* (Supporting Information, Appendix 1).

The sociodemographic questions included were respondents’ sex, age, educational status, religion, marital status, socioeconomic status, livelihood practice, livestock ownership, farming system and districts. Age class of the respondents was classified into three groups as less than 30 years, 30–45 years and greater than 45 years following previous recommendation for social science research (Nigatu Yalemebrat et al. 2016; Dinbiso et al. 2025). The questionnaires were pilot tested on 15 respondents, and amendment was made accordingly. The pilot testing was primarily targeted to test the clarity of questions: additionally, to estimate the time needed to administer the questions without making any distress for respondent and enough to acquire the required information. The questionnaire was originally prepared in English, and then it was translated to local language, Amharic, and then back to English by an external translator to check for consistency. The Amharic version of the questionnaire was administered by face‐to‐face interviews. Interviews were conducted at the household level. Each interview lasted approximately 30–45 min.

### 2.3 Sampling Strategy and Sample Size Determination

A multistage sampling technique was employed to select representative participants from different districts to ensure the representation of various agroecological zones and farming systems. This approach allowed for a comprehensive assessment of the KAP regarding livestock diseases across diverse contexts within the East Gojjam Zone. First, the five districts were selected purposively based on livestock population density and ease of accessibility. Next, *kebeles* within districts, village within kebele households within villages were selected randomly. List of *kebeles* was obtained from the district agricultural office, and list of villages and households was obtained from the *kebele* administrative offices. Households were selected randomly using computer‐based random number. Individuals within households were selected purposively targeting household head; however, participants with age greater than 18 years of old were substituted when the head of the household was not available. Household in the vicinity were substituted when no one was available or unwilling to participate from the targeted household. Key informants, including local veterinary officers and development agents, were also purposively selected for key informant interviews to enrich the quantitative data with expert perspectives on livestock disease prevalence, control measures and community engagement.

The required sample size for the quantitative household survey was determined using a single population proportion (Cochran) formula, assuming a 50% proportion of adequate knowledge about livestock diseases (due to lack of prior data), a 95% confidence level and a 5% margin of error (Thrusfield 2018). *n* = $\frac{\;z^{2}\;p\left(1-p\right)}{d^{2}}$, where *n* = required sample size, *z* = value for selected alpha level of 0.025 in each tail = 1.96, *p* = estimated of proportion of an attribute presented in the study population and *d* = acceptable margin of sampling error for mean being estimated. Based on the formula, the number of households required is 384. By considering ten percent non‐response rate for the questionnaire survey, the required total number of households reached 423. The sample size was distributed among the administrative districts proportional to population size. But during the questionnaire interview, about 11 respondents did not give complete information, and it was removed in the final analysis; then only 412 respondents were used.

## 3 Data Management and Statistical Analysis

The responses for KAP questions were given scores and dichotomized using cut off 50% of the maximum obtainable score, reflecting good and poor KAP value. The binary knowledge questions were given a score of 1 when correctly answered, and 0 when answered incorrectly. Respondents who scored greater than or equal to 50% were considered to have adequate knowledge and those who scored less than 50% were considered to have inadequate knowledge. Similarly, responses for practice were scored 1 as (has good practice) and 0 (has poor practice). Respondents who have scored 50% and above were categorized to have good practice and individuals who scored less than 50% to have poor practice.

The attitude questions were set in a Likert scale and were scored 1 = *strongly agree*, 2 = *agree*, 3 = *uncertain*, 4 = *disagree*, 5 = *strongly disagree* or in reverse 1 = *strongly disagree*, 2 = *disagree*, 3 = *uncertain*, 4 = *agree* and 5 = *strongly agree* depending on the nature of the statement. In both cases, the high numbered responses were given towards the desirable direction. A person`s attitude was determined based on the sum of the values given for questions measuring attitude and made binary variable in to desirable attitude (for attitude score ≥ 50%) and undesirable attitude (for attitude score < 50%; Bahiru et al. 2022). Sociodemographic characterization and distribution knowledge, attitude and practice of the participants were summarized using descriptive statistics. Mixed effect logistic regression model was used to determine the association of the livestock disease with the potential risk factors towards adequate knowledge, good practice and desirable attitude. Sex, age category, educational status, religion, marital status, income level, livestock ownership, livelihood practice, farming system and districts were the predictor variables where associations were examined. Factors with the *p*‐value less than 0.25 in the univariable analysis were incorporated into the full multivariable logistic regression model. In the multivariable logistic regression, *p*‐value < 0.05 was considered as cut off for statistical significance, and adjusted odd ratio (AOR) and 95% CI were also calculated. Multicollinearity test was done before fitting the variable in to multivariable regression model to rule out a significant correlation between the predictor variables. Confounding and interactions were checked for the variables in the final model. Model validation was done using the standard Hosmer and Lemeshow test and deviance (Boateng and Abaye 2019). The data were extracted using a standardized data extraction format created in Microsoft Excel, and the analysis was performed using STATA‐17 statistical software (StataCorp 1985, 2013).

## 4 Results

### 4.1 Sociodemographic Characteristics of the Respondents

During the survey period, a total of 412 participants who were approached successfully completed the questionnaire. The majority of respondents (88%) were male and married, and 51% belonged to the adult age group (30–45 years). Only 8% of participants had attained an education at the college level or higher. Furthermore, 88% of respondents were engaged in mixed crop–livestock farming. Regarding household income, only 15% reported having an intermediate income level (6000–10,000 ETB). Most respondents identified as Orthodox Christians. Approximately 83% owned livestock, with only 6% reporting ownership of cattle species exclusively (Table 1).

**TABLE 1:** Sociodemographic characteristics of respondents (*N* = 412) in selected districts of East Gojjam Zone, Amhara Region, Ethiopia.

			Mean score		
Variable	Category	Number (%)	Knowledge (SD)	Attitude (SD)	Practice (SD)
Sex	Male	363 (88)	0.57 (0.15)	0.69 (0.07)	0.50 (0.14)
	Female	49 (12)	0.56 (0.13)	0.67 (0.08)	0.48 (0.16)
Age	Youth (≤ 30 years)	74 (18)	0.59 (0.14)	0.69 (0.09)	0.49 (0.14)
	Adult (30–45 years)	193 (47)	0.56 (0.15)	0.68 (0.06)	0.49 (0.14)
	Old (≥ 46 years)	145 (35)	0.57 (0.15)	0.69 (0.07)	0.50 (0.16)
Level of education	Illiterate	198 (48)	0.57 (0.14)	0.68 (0.07)	0.49 (0.15)
	Primary school (1–8 grade)	138 (34)	0.57 (0.15)	0.68 (0.08)	0.51 (0.15)
	Secondary school (9–12)	43 (10)	0.58 (0.17)	0.69 (0.07)	0.49 (0.14)
	College and above	33 (8)	0.56 (0.14)	0.71 (0.05)	0.50 (0.13)
Religion	Orthodox Christians	378 (92)	0.57 (0.15)	0.68 (0.07)	0.50 (0.15)
	Muslims	30 (7)	0.53 (0.15)	0.69 (0.08)	0.49 (0.12)
	Others	4 (1)	0.57 (0.12)	0.73 (0.01)	0.31 (0.08)
Marital status	Married	361 (88)	0.57 (0.15)	0.69 (0.07)	0.50 (0.14)
	Divorced	18 (4)	0.59 (0.14)	0.66 (0.07)	0.48 (0.19)
	Single	19 (5)	0.58 (0.15)	0.71 (0.07)	0.49 (0.15)
	Windowed	14 (3)	0.57 (0.12)	0.68 (0.04)	0.50 (0.18)
Household income level (ETB)	(< 6000)	333 (81)	0.57 (0.15)	0.69 (0.07)	0.50 (0.14)
	Intermediate (6000–10,000)	61 (15)	0.59 (0.16)	0.68 (0.08)	0.50 (0.15)
	High (> 10,000)	18 (4)	0.57 (0.13)	0.69 (0.07)	0.44 (0.17)
Livelihood practice	Crop production	15 (4)	0.59 (0.13)	0.69 (0.05)	0.52 (0.12)
	Livestock production	19 (5)	0.60 (0.17)	0.71 (0.06)	0.55 (0.16)
	Trading	13 (3)	0.55 (0.18)	0.70 (0.07)	0.45 (0.11)
	Mixed (crop–livestock)	365 (88)	0.57 (0.15)	0.68 (0.07)	0.49 (0.15)
Farming system	Extensive	269 (65)	0.53 (0.14)	0.64 (0.06)	0.48 (0.14)
	Semi‐intensive	143 (35)	0.66 (0.11)	0.66 (0.06)	0.52 (0.15)
Livestock ownership	Yes	344 (83)	0.57 (0.14)	0.69 (0.07)	0.49 (0.14)
	No	68 (17)	0.55 (0.16)	0.67 (0.07)	0.50 (0.16)
Districts	Awabel	71 (17.2)	0.52 (0.15)	0.68 (0.07)	0.48 (0.15)
	Enemay	84 (21.4)	0.63 (0.13)	0.68 (0.08)	0.50 (0.16)
	Goncha siso enesie	84 (20.4)	0.53 (0.14)	0.70 (0.05)	0.51 (0.14)
	Gozamen	90 (21.8)	0.60 (0.15)	0.68 (0.07)	0.48 (0.15)
	Hulet eju enesie	83 (20.1)	0.56 (0.14)	0.69 (0.08)	0.50 (0.12)
Mean overall score		412 (100)	0.57 (0.15)	0.69 (0.07)	0.49 (0.14)

### 4.2 Mean Score of Knowledge, Attitude and Practice of Respondents

The mean scores for knowledge, attitude and practice related to livestock diseases were 57%, 69% and 49%, respectively (Table 1). Overall, 72.6% of participants demonstrated adequate knowledge, 97.3% exhibited a desirable attitude and 47.3% reported good practices regarding livestock diseases, their management and preventive measures.

### 4.3 Respondents’ Knowledge Towards Livestock Disease

All respondents demonstrated awareness of livestock diseases and their economic significance. Approximately 96% identified feed shortages and unrestricted grazing as primary constraints to livestock production, followed by diseases (92%). With regard to major cattle diseases, all participants recognized anthrax, while 98% and 97% were aware of tick infestations and fasciolosis, respectively. Similarly, all respondents recognized fasciolosis as a major disease affecting sheep and goats, followed by other gastrointestinal parasitic infections (98%) and contagious ecthyma (Orf or mouth sore; 97%). Furthermore, 98% of respondents reported that loss of appetite is a characteristic clinical sign of disease in livestock, and 72% indicated that they could differentiate between diseased and healthy animals based on clinical presentation. Awareness of zoonotic diseases was relatively limited; only 45% of participants mentioned the potential for disease transmission from animals to humans. Moreover, 85% identified ingestion of contaminated water or feed as a principal source of infection. Approximately 56% of respondents acknowledged that markets could serve as sources of disease transmission. While 64% of participants recognized vaccination as an important strategy for disease control and prevention, only 45% demonstrated knowledge of appropriate vaccination intervals for livestock (Table 2).

**TABLE 2:** Summary of knowledge among respondents regarding livestock diseases in selected districts of East Gojjam Zone, Amhara Region, Ethiopia (*n* = 412).

Livestock disease knowledge question/item	Number of correct and/yes respondents (%)
Awareness of livestock disease	412 (100)
Livestock production constraints:	
Feed and free grazing shortage	397 (96)
Disease	380 (92)
Low genetic potential	50 (12)
Poor management (extension support)	30 (7)
Common cattle diseases identified	
Anthrax	412 (100)
Fasciolosis	400 (97)
Lumpy skin disease	300 (73)
Black leg	325 (79)
Foot and mouth disease	250 (61)
Skin disease/dermatitis(tick)	403 (98)
Gastrointestinal parasitism	150 (36)
Pneumonia/pasteurellosis	70 (17)
Mastitis	210 (51)
Trypanosomiasis	20 (5)
Common sheep and goat disease	
Shoat pox	384 (93)
Orf (mouth sore)	399 (97)
Fasciolosis	412 (100)
Coenuruses/circling disease	320 (78)
Anthrax/sudden death/cowdrosis	30 (7)
Skin disease/dermatitis (sheep ked, flea and tick)	290 (70)
Pest des petties ruminants (PPR)	153 (37)
Gastrointestinal parasitism	405 (98)
Clinical signs to differentiate sick from healthy animals	
Loss of appetite	402 (98)
Weakness	390 (95)
Cough	388 (94)
Diarrhoea	397 (96)
Poor body condition/unthriftiness	322 (78)
Rough hair coat	198 (48)
Sudden death	212 (51)
Could you identify diseased animal from healthy one	296 (72)
Zoonotic knowledge	
Know diseases can be transmitted from animal to human	183(45)
Know diseases can be transmitted from human to animal	165 (40)
Sources of transmission identified	
Ingestion	352 (85)
Contact	251 (61)
Air‐borne and water‐borne	300 (73)
Sexual transmission	15 (4)
Inoculation/during treatment	9 (2)
Knowledge on control and prevention	
Know disease control methods for sick animals	390 (95)
Disease prevention and control methods	
Vaccination	264 (64)
Prophylaxis/deworming	133 (32)
Quarantine	111 (27)
Isolation/movement restriction	39 (9)
Culling/stamping out	32 (8)
Know the availability of vaccines and animal drugs	283 (69)
Believe all livestock diseases are preventable by vaccination	176 (43)
Know vaccination intervals	185 (45)

### 4.4 Participant Attitude Towards Livestock Disease

The majority of participants (58%) agreed that livestock diseases have significant economic and zoonotic importance. Additionally, 62% of respondents believed that the spread of animal diseases could be prevented. Among them, 69% agreed that regular vaccination and prophylactic measures are effective strategies for livestock disease prevention. However, only 43% of respondents expressed willingness to vaccinate their animals; and 36% reported dissatisfaction with the coverage of vaccination campaigns provided in their area. About 58% of respondents disagreed that disease outbreaks were properly handled and managed by animal health care providers within their communities. Furthermore, 38% of respondents disagreed with the statement that traditional healers can effectively treat animal diseases. Only 36% of participants strongly agreed that they actively sought information regarding disease outbreaks (Table 3).

**TABLE 3:** Summary of attitude among respondents of selected districts of East Gojjam Zone, Amhara, about livestock disease in Ethiopia (*n* = 412).

	Response, number (%)				
Livestock disease attitude question	Strongly agree	Agree	Not sure (uncertain)	Disagree	Strongly disagree
Livestock disease has significant economic and zoonotic importance	103 (25)	240 (58)	42 (10)	27 (7)	−
Spread of livestock disease can be prevented	40 (10)	257 (62)	71 (17)	42 (10)	2 (1)
Regular vaccination and prophylactic measures are effective for disease prevention	62 (15)	284 (69)	42 (10)	19 (5)	5 (1)
Livestock vaccine and drug is affordable	24 (6)	117 (28)	120 (29)	151 (37)	−
Willingness to vaccinate and prophylaxis animals	156 (38)	177 (43)	38 (9)	31 (8)	10 (2)
Satisfaction with vaccination campaign coverage	26 (6)	115 (28)	123 (30)	147 (36)	1 (0.01)
Cost of vaccination is factor for vaccination campaign	8 (2)	39 (9)	75 (18)	251 (61)	39 (10)
Report of sick or dead animals to the local veterinary authorities/officers	23 (5)	238 (58)	65 (16)	81 (20)	5 (1)
Outbreaks are properly handled by animal health care providers	18 (5)	55 (13)	90 (22)	240 (58)	9 (2)
Traditional healers can effectively treat animal disease	2 (0.0)	97 (24)	143 (35)	156 (38)	14 (3)
Actively seek information about disease outbreaks	150 (36)	133 (32)	65 (16)	27 (7)	37 (9)

### 4.5 Participant Practice Towards Livestock Disease

Approximately 84%, 68% and 43% of respondents reported that they treated their sick animals, avoided using meat from sick animals for consumption and refrained from drinking raw milk from sick animals, respectively. Only 42% of respondents separated sick animals from healthy ones. Moreover, only one‐third (32%) reported using personal protective equipment (PPE) when handling sick animals. About 82% preferred modern veterinary treatments, while 59% indicated that they used botanical remedies to treat their sick animals. Additionally, only 30% and 38% of respondents reported vaccinating and deworming their animals, respectively, as a disease prevention strategy. A minority of respondents (21%) disposed of dead animals properly through burial or burning (Table 4).

**TABLE 4:** Summary of practices among respondents in selected districts of East Gojjam Zone, Amhara Region, regarding livestock diseases in Ethiopia (*n* = 412).

Livestock disease practice question	Number of correct and/yes of respondents (%)
Separated diseased animals from healthy ones	172 (42)
Avoided slaughtering sick animals for meat consumption	282 (68)
Avoid drinking raw milk from sick animals	178 (43)
Satisfactory veterinary service in your area	187 (45)
Treated animals when sick	346 (84)
Used personal protective equipment when handling sick animals	133 (32)
Type of medical treatment sought	
Traditional	74 (18)
Modern	338 (82)
Used botanical remedies for treating sick animals	242 (59)
Vaccinated animals against disease	124 (30)
Used deworming and prophylaxis	155 (38)
Avoided contact with neighbouring animals to reduce disease	134 (33)
Methods of disposing dead animals	
Threw carcass on the street	298 (72)
Burial	86 (21)
Burning	4 (1)

### 4.6 Livestock Demographic Characteristics and Their Management

**FIGURE 2:**
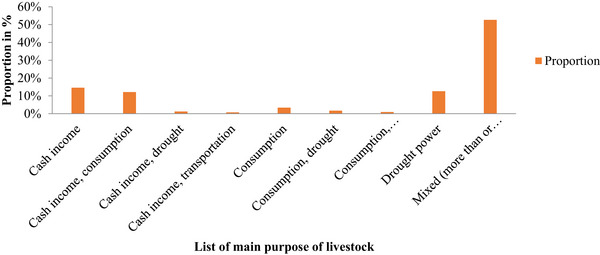
Purpose of Livestock keeping among respondents in selected areas of East Gojjam Zone, Ethiopia (*n* = 412).

The majority of respondents (83.5%) owned one or more species of livestock. Over half (51%) reported a decreasing trend in livestock population, and 71% indicated that adult animals constituted the dominant age group. Approximately 53% of participants described their livestock as serving multiple purposes (three or more; Figure 2).

Regarding management practices, 77% of respondents stated that all family members were involved in animal care, while only 42% reported that their animals relied on private sources for water and feed. Furthermore, 87% of participants housed their livestock in house barn‐type shelters. Livestock acquisition was primarily through purchase as indicated by 36% of respondents (Table 5).

**TABLE 5:** Demography and management practice of animal in East Gojjam of Amhara Region, Ethiopia.

Variable	Category	Respondents’ frequency (%)
Dominant animal age	Young	58 (14)
	Adult	293 (71)
	Old	61 (15)
Who care for the animals	All	317 (77)
	Father	49 (12)
	Mother	31 (7)
	Children	15 (4)
Housing type	House barn	359 (87)
	Fenced	53 (13)
Feed and water source	Communal	225 (55)
	Communal and private	12 (3)
	Private	175 (42)
Source of animal	Gift	28 (7)
	Gift and purchase	113 (27)
	Purchase	148 (36)
	Restocking	123 (30)
Over all		**412 (100)**

### 4.7 Factors Associated With Participant's Knowledge, Attitude and Practice Towards Livestock Disease

Sex, age category, educational level, livestock ownership and the respondent farming system showed a significant association at a *p*‐value of 0.25 in univariable analysis and included in the multivariable logistic regression model. In the multivariable logistic regression model, all of the variables except the sex of respondents were associated with knowledge of the respondents (Table 6). The attitude multivariable logistic regression model only includes the age category of the respondents as the predictor variable associated with respondents’ attitude for livestock disease. Respondents’ youth age category (≤ 30 years) were 0.09 (95%CI: 0.02–0.52, *p* = 0.007) times less desirable attitude towards livestock disease than adult age (between 30 ≤ 45) years respondents. District, sex, educational level, marital status, livelihood practice and the respondent farming system showed a significant association at a *p*‐value of 0.25 in univariable analysis and were incorporated in the multivariable logistic regression model. All of these variables except educational level were not predictor variables and not significantly associated with the practice level of respondents in the multivariable logistic regression model (Table 7).

**TABLE 6:** Logistic regression model that shows factors associated with knowledge about the livestock diseases among respondents in East Gojjam, Ethiopia (*n* = 412).

Variable	Category	Adequate knowledge (*n* = 299)		Adequate knowledge (*n* = 299)	
		Univariable		Multivariable	
		COR (95% CI)	*p*‐value	AOR (95% CI)	*p‐*value
Sex	Female	Ref.	−		
	Male	1.79 (0.97–3.3)	0.067		
Age	Adult	Ref.	−	Ref.	−
	Youth	0.09 (0.48–1.6)	0.686	0.95 (0.49–1.86)	0.886
	Old	0.58 (0.36–0.93)	0.024	0.58 (0.35–0.96)	0.035
Educational level	College and above	Ref.	−	Ref.	−
	Illiterate	0.27 (0.08–0.90)	0.035	0.24 (0.06–0.87)	0.030
	Primary school	0.22 (0.06–0.76)	0.017	0.18 (0.048–0.65)	0.009
	Secondary school	0.21 (0.05–0.79)	0.022	0.15 (0.37–0.63)	0.009
Livestock ownership	No	Ref.	−	Ref.	−
	Yes	1.9 (113–3.35)	0.016	2.32 (1.29–4.17)	0.005
Farming system	Extensive	Ref.	−	Ref.	−
	Semi‐intensive	3.85 (2.22–6.70)	0.000	4.15 (2.34–7.36)	0.000

*Note*: AOR: adjusted odds ratio; COR: crude odds ratio; Ref: reference.

**TABLE 7:** Logistic regression model that shows factors associated with practice about the livestock diseases among respondents in East Gojjam, Ethiopia (*n* = 412).

Variable	Category	Good practice (*n* = 195)		Good practice (*n* = 195))	
		Univariable		Multivariable	
		COR (95% CI)	*p*‐valu*e*	AOR (95% CI)	*p‐*value
Districts	Awabel	Ref.	−		
	Enemay	0.69 (0.37–1.32)	0.272		
	Goncha siso enesie	1.51 (0.8–2.9)	0.203		
	Gozamen	0.6 (0.32–1.12)	0.168		
	Hulet eju enesie	1.12 (0.6–2.1)	0.756		
Sex	Female	Ref.	−		
	Male	1.8 (0.97–3.38)	0.062		
Educational level	College and above	Ref.	−	Ref.	−
	Illiterate	0.04 (0.01–0.17)	0.000	0.041 (0.009–0.19)	0.000
	Primary school	0.04 (0.01–0.19)	0.000	0.042 (0.009–0.2)	0.000
	Secondary school	0.21 (0.04–1.04)	0.057	0.23 (0.042–1.27)	0.092
Marital status	Divorced	Ref.	−		
	Married	3.3 (1.05–10.1)	0.041		
	Single	4.8 (1.14–20.24)	0.032		
	Windowed	2.6 (0.57–12.18)	0.218		
Livelihood practice	Crop production	Ref.	−		
	Livestock production	4.3 (0.96–19.2)	0.057		
	Mixed LC	0.97 (0.35–2.74)	0.961		
	Trading	0.7 (0.16–3.23)	0.662		
Farming system	Extensive	Ref.	−		
	Semi‐intensive	1.3 (0.87–1.97)	0.191		

*Note*: AOR: adjusted odds ratio; COR: crude odds ratio; Ref: reference.

## 5 Discussion

Livestock diseases emerged as a major concern among respondents in East Gojjam Zone, reflecting a broader pattern observed throughout Ethiopia and other low‐income countries with significant livestock populations. In East Gojjam, the diverse agroecological zones and climatic variability create conditions favourable for the proliferation of a wide range of pathogens, including those responsible for endemic and emerging diseases. This study provides important insights into farmers’ KAP related to livestock diseases and their management in the East Gojjam Zone of the Amhara Region, Ethiopia. The findings indicate that the majority of respondents demonstrated good knowledge and a positive attitude towards livestock diseases and their control measures. However, their actual preventive practices were notably poor. This study found that 72.6% of respondents were found to possess adequate knowledge about common livestock diseases, their symptoms and the importance of disease prevention. These findings are consistent with those reported by Tufa et al. (2023), who observed similar patterns of high awareness and favourable attitudes but limited implementation of best practices in livestock health management. The gap between knowledge and practice may be attributed to structural barriers such as limited access to veterinary services, lack of resources to implement preventive measures (e.g., regular vaccinations, isolation of sick animals) and weak extension support. These findings align with the studies by Robi et al. (2023) and Makarabbi et al. (2025), which highlighted that despite possessing knowledge, livestock owners often do not translate this into effective practices due to these systemic challenges.

In the current study, approximately 97.3% of respondents exhibited a desirable attitude towards livestock diseases and their management. This finding is consistent with previous reports, such as Bahiru et al. (2022), and even surpasses the positive attitude rate of 80% reported by Tufa et al. (2023). The higher proportion observed in East Gojjam may suggest a growing awareness and concern among rural livestock keepers, indicating a potentially favourable environment for the implementation of control measures provided that appropriate support is available from veterinary and extension services.

Despite favourable knowledge and attitudes, only 47.3% of respondents demonstrated good practices in managing livestock diseases. This is consistent with findings from Bahiru et al. (2022) and Tufa et al. (2023) but lower than reported in Mekelle (61.3%; Hagos et al. 2020) and Nigeria (74%; Edukugho et al. 2018). The relatively low level of preventive practices in this study may be attributed to several contextual factors. The majority of respondents in East Gojjam are from rural areas, often with limited education, and primarily engaged in crop or mixed farming systems. In contrast, participants in studies reporting higher practice scores were typically urban‐based, more educated and more specialized in livestock production. These differences suggest that structural and educational barriers may significantly influence the translation of knowledge and attitudes into concrete disease management practices.

### 5.1 Respondents’ Knowledge Towards Livestock Disease

The current study revealed that the majority of respondents possessed a good understanding of common livestock diseases prevalent in their area. This observation aligns with the findings of Tafa et al. (2023), which similarly reported high levels of awareness among rural livestock owners. The results suggest that local farmers in East Gojjam have developed substantial indigenous knowledge and skills related to animal health, likely shaped by generations of experience and observation. While most respondents demonstrated an awareness of livestock diseases and their economic implications, the study also uncovered a critical knowledge gap concerning zoonotic disease transmission. Only 44.5% of participants recognized that diseases can be transmitted between animals and humans, a figure comparable to the 45.1% reported by Abunna et al. (2024). This limited awareness of zoonoses poses public health risks, particularly in communities where close human–animal interactions are common, and hygiene practices may be insufficient.

Variations in knowledge levels across different regions and respondent groups may be influenced by factors such as age, education level and access to information through radio, television or mobile platforms. Similar conclusions were drawn by Amenu et al. (2010), who found that educational background and media exposure significantly affected farmers' awareness of zoonotic diseases.

#### 5.1.1 Perceived Prevalent Livestock Diseases

In this study, the most frequently reported livestock diseases affecting cattle were anthrax, skin diseases including mange and tick infestation, fasciolosis, blackleg and LSD. These findings reflect local disease perception patterns in the East Gojjam Zone but contrast with reports from other regions of Ethiopia. For instance, Haftu et al. (2014) identified FMD, pasteurellosis, ectoparasite (tick) infestations and anthrax as the most common diseases in Ganta Afeshum Woreda, northern Ethiopia. Similarly, Tessema and Negash (2022) reported mastitis, bovine pasteurellosis, cowdriosis and external parasites (ticks) as the major cattle health issues in Arbegona District, Sidama Region.

Regarding sheep and goats, participants in the current study identified fasciolosis, gastrointestinal parasitism and contagious ecthyma (ORF) as the most prevalent diseases. This aligns with findings from Haftu et al. (2014), who also reported fasciolosis and ORF as major small ruminant diseases in Ganta Afeshum. However, the current results differ slightly from those of Abraha (2007), who highlighted coenuruses as the predominant problem in sheep and goats in the Atsebi‐Wonberta area, and from Tessema and Negash (2022), who reported ovine pasteurellosis as the top‐ranked disease in the Arbegona District of Sidama Region. Interestingly, the identification of contagious ecthyma (ORF) as a major problem in the present study closely matches findings by Bayeleyegn (2007), who also noted ORF as a key disease of small ruminants. These discrepancies across regions may stem from differences in agro‐ecological conditions, livestock management systems and disease exposure patterns. Variations in reporting may also reflect differences in farmers' experiences, access to veterinary diagnosis and levels of awareness.

The findings of this study indicate that nearly 95% of respondents were aware of various livestock disease control methods, demonstrating a generally positive attitude towards disease management. Among these methods, vaccination was identified as the primary preventive strategy by approximately 64% of respondents. This aligns with the findings of Tessema and Negash (2022) and Tufa et al. (2023), who also reported vaccination as the most widely recognized and practised disease control method among livestock owners. However, despite recognising vaccination as a critical intervention, only 45% of the respondents had adequate knowledge about the appropriate intervals and application of vaccines. This partial understanding may reduce the effectiveness of disease prevention efforts, as improper timing or administration can compromise vaccine efficacy. A similar issue was noted by Haftu et al. (2014), where 68.3% of respondents reported relying solely on vaccination but lacked comprehensive knowledge about its proper implementation. Overall, the community demonstrates a positive attitude and an openness to modern veterinary practices, including the use of vaccines and other medical treatments. Consistent with findings from the Arbegona District (Tessema and Negash 2022), many farmers in East Gojjam reported visiting veterinary clinics when their animals became ill, while others resorted to administering modern medicine on their own. These behaviours suggest a willingness to engage with veterinary services, although gaps in technical knowledge remain.

### 5.2 Respondents’ Attitude and Practice Towards Livestock Disease

In this study, nearly 68% of the participant farmers reported that they did not consume or use animal products, such as meat and milk, from sick or dead animals. This finding is significant, as it suggests that a considerable proportion of the respondents are aware of the potential health risks associated with consuming animal products from animals that are sick or have died. Despite this awareness, over 85% of the respondents also recognized that raw meat and milk could serve as vehicles for disease transmission, indicating that many participants understand the zoonotic risks involved. However, the discrepancy between knowledge and practice is noteworthy. The findings align with the study by Abunna et al. (2024), which showed that 75.1% of respondents avoided consuming animal products from sick or dead animals. This consistency in results across different studies suggests that a general awareness about the risks of consuming contaminated animal products exists within Ethiopian communities. However, despite this awareness, many farmers continue to consume raw milk, either fresh or fermented, which is common in Ethiopian society. This trend indicates a lack of understanding regarding the specific zoonotic risks associated with raw milk consumption, such as the potential transmission of diseases like brucellosis or tuberculosis. Raw milk consumption, both fresh and fermented, remains widespread in Ethiopia, even among farmers who are aware of its potential risks. In a similar study conducted in the southwestern part of Ethiopia, 82.3% of respondents were aware of the risks associated with consuming raw or undercooked meat, particularly regarding taeniasis, a parasitic infection. This further highlights the awareness of zoonotic risks in certain regions of Ethiopia. However, there is considerable variation between regions, with some farmers more likely to be aware of these risks than others. The differences in knowledge and practices can likely be attributed to various factors, including educational attainment, media access and local disease prevalence.

Regarding veterinary services and their coverage, this study found that 55% of respondents expressed dissatisfaction with the availability and adequacy of veterinary services in their area. This dissatisfaction is linked to the perception that the coverage of veterinary services is insufficient to meet their needs. A similar study conducted by Haftu et al. (2014) in the Gantaafeshum woreda of northern Ethiopia found that 75% of respondents reported a shortage of animal health centres in their region. The reasons for this shortage were twofold: the distance of available clinics from respondents’ residences and the lack of adequate facilities within the clinics to provide proper veterinary care. These findings suggest that access to veterinary services in rural Ethiopia is limited by both physical distance and insufficient infrastructure, which impacts the effectiveness of the available services. Further supporting this concern, Moges and Bogale (2012) found that only 41.9% of respondents in the Lay‐Armacheho District, northwestern Ethiopia, had access to modern veterinary services, leaving 58.1% without access to these critical services. This disparity underscores the challenges in accessing veterinary care and highlights the gaps that exist in the delivery of these essential services, particularly in remote areas. The findings from these studies, including the current one, suggest that the East Gojjam Zone faces similar limitations in terms of veterinary services. The lack of veterinary service infrastructure is a critical issue that can lead to inadequate animal health management, ultimately affecting livestock productivity and contributing to the spread of diseases. These challenges are consistent with reports from the zonal agricultural office, which further emphasize the need for improved veterinary service coverage and accessibility.

Furthermore, a significant proportion of participants (68%) in this study reported that they had never used PPE when handling sick animals. This finding is consistent with previous studies in Ethiopia, such as the one by Abunna et al. (2024), where 77.3% of respondents similarly indicated that they did not use PPE in similar situations. The lack of PPE usage in these areas can primarily be attributed to its scarcity, as farmers and pastoralists often do not have access to the necessary protective gear, especially when dealing with animal‐related health issues, such as abortion. Deneke et al. (2022) highlighted that due to this scarcity, many farmers resort to handling sick animals without any protection, which significantly increases their risk of exposure to zoonotic diseases. The absence of protective gear, coupled with a lack of awareness and resources, further exacerbates the risk for farmers and their families.

Most of the respondents in this study were aware of various disease control and prevention methods available for livestock, with 82% of them expressing a preference for implementing modern approaches to prevent and treat diseases in exposed livestock. This indicates that a significant proportion of farmers are open to adopting contemporary veterinary practices to manage livestock health. However, despite the general preference for modern approaches, an interesting paradox emerged; 81% of respondents believed that traditional treatments were more effective for managing livestock diseases. This reliance on traditional treatments presents a challenge, as the effectiveness of many traditional remedies is not scientifically established, and their use may not always address the underlying health issues that livestock face (McCorkle 1986, Abraha 2016). The discrepancy between knowledge and practice is concerning, as it suggests that even though farmers may recognize the value of modern veterinary practices, they continue to rely heavily on traditional treatments. This may be due to a variety of factors, including cultural beliefs, accessibility to modern veterinary services or a lack of trust in modern treatments. The findings from this study align with the report by Bahiru et al. (2022), which highlighted similar patterns in the use of traditional treatments despite an awareness of modern medical options.

The majority of farmers in this study (52%) reported that they had never dewormed their animals. This finding highlights a significant gap in the routine health management practices for livestock, as deworming is an essential practice for preventing parasitic infections that can negatively impact the health and productivity of animals. The lack of deworming could lead to increased susceptibility to diseases, lower livestock productivity and potential zoonotic risks for humans. This result is consistent with findings from a similar study conducted in Ada'a district, Oromia, Ethiopia, where 48.3% of respondents reported never having dewormed their animals (Abunna et al. 2024). This suggests that the practice of regular deworming is not widely adopted among farmers in various regions of Ethiopia, even though it is a crucial component of livestock management. The reasons for not deworming animals may include a lack of awareness about the importance of deworming, limited access to veterinary services, or financial constraints. In some cases, farmers may not recognize the long‐term benefits of deworming or may prioritize other aspects of animal care. Additionally, the availability and affordability of deworming medications may be barriers in rural areas.

In this study, a significant proportion of respondents (72%) reported that they discarded dead animals by leaving them in the surroundings for wild and domestic canines to scavenge. This practice poses a serious public health risk, as the carcasses of dead animals can be a source of disease transmission. When canines, both wild and domestic, have access to these carcasses, they can spread zoonotic diseases to other animals and even humans. Pathogens from the dead animals may also contaminate the environment, leading to further outbreaks of infectious diseases. The habit of improper carcass disposal is not isolated to this study, as it has been reported in several regions of Ethiopia. For example, Bahiru et al. (2022) also highlighted this practice as a significant risk factor for disease transmission. Furthermore, similar studies have shown that improper disposal of livestock carcasses is a common issue in rural areas of many developing countries, where access to proper disposal methods is limited (Beyene et al. 2018). Studies have linked improper carcass disposal to the spread of diseases such as rabies, anthrax and brucellosis (Ackah 2020), all of which can be transmitted through contact with infected carcasses or via scavenger animals. The act of discarding carcasses in open areas further exacerbates the potential for disease transmission, especially in areas where proper disposal methods are not practised. Given the risks associated with this practice, it is crucial to educate communities about the importance of proper carcass disposal. Safe disposal methods such as deep burial, incineration or transporting carcasses to designated disposal sites can significantly reduce the risk of disease spread (Vithanage et al. 2021).

### 5.3 Livestock Ownership and Management Practices

The findings of this study provide valuable insights into livestock ownership and management practices among households in East Gojjam, Amhara Region. The high rate of livestock ownership (83.5%) confirms the central role of livestock in rural livelihoods, serving as a source of income, food, draught power and social status. This is consistent with previous studies conducted in other parts of Ethiopia, such as in Oromia and Tigray, where livestock ownership was similarly widespread and multifunctional (Tegegne et al. 2013; Gizaw 2010). Regarding livestock use, over half of the respondents (53%) indicated that animals served multiple purposes. This multifunctional role of livestock has been well‐documented across Ethiopia and other parts of sub‐Saharan Africa, where livestock are vital not only for direct outputs like milk and meat but also for indirect benefits such as manure, traction and asset accumulation (Catley et al. 2013; Turk 2013).

The fact that 51% of respondents reported a decline in livestock population may reflect growing challenges such as feed shortages, recurrent droughts, disease outbreaks and economic pressures. Similar concerns were reported in studies from southern Ethiopia, where livestock holdings were affected by climate variability and rangeland degradation (Tegegn et al. 2018; Yusuf 2021; Tsegaye et al. 2013). The dominance of adult animals (71%) within herds suggests a prioritization of productive animals that contribute to household income and agricultural labour. This age distribution aligns with findings from central and southern Ethiopia, where adult cattle are retained for milk, draft and breeding purposes (Anteneh 2006).

Management practices observed in this study further reflect traditional norms and resource limitations. The high level of family involvement in animal care (77%) is in line with findings from studies in the Amhara and Afar regions, where livestock duties are shared among household members due to limited access to hired labour (Etefa 2020). The reliance on house barn‐type shelters (87%) is also consistent with traditional housing practices aimed at protecting animals from theft, predation and harsh weather (Tariku et al. 2025). The use of communal feed and water sources (55%) poses potential risks for disease transmission and resource conflicts. Similar trends have been noted in highland Ethiopia, where competition for communal resources often exacerbates livestock stress during dry seasons (Deressa et al. 2010). The fact that 42% of respondents rely on private resources may indicate a shift towards more controlled and sustainable livestock management. The finding that livestock acquisition is primarily through purchase (36%), followed by restocking programmes (30%), reflects both market activity and external support mechanisms. This pattern has also been observed in post‐drought recovery programmes in pastoral and mixed farming areas (Yilma et al. 2011), suggesting the importance of accessible markets and timely aid interventions.

### 5.4 Factors Associated With Participant's Knowledge, Attitude and Practice Towards Livestock Disease

There is a statistically significant difference in knowledge scores between the education level of the respondents, where respondents with a college and above education background have a higher knowledge of livestock disease, compared to illiterates and those with only a primary school education. This finding aligns with previous studies, where participants with higher education levels were more likely to possess adequate knowledge about livestock diseases (Bahiru et al. 2022; Ameh et al. 2014; Costa and Fernandes 2016; Guadu et al. 2014). Education likely provides better access to information and enhances the ability to comprehend the nature of diseases and health education materials. Educated individuals tend to be more exposed to formal information sources such as health programmes, media and agricultural advisory services, which improve their understanding of livestock health management. In addition to education, age was also found to be a significant factor influencing knowledge levels. The study shows that adult respondents (31–45 years) were more likely to have better knowledge of livestock diseases, compared to younger individuals (under 30 years). This finding is consistent with a report from the North Shewa Zone of Ethiopia (Legesse et al. 2022). The increased knowledge among adults may be attributed to their longer exposure and experience with livestock production, giving them a deeper understanding of animal diseases over time. Moreover, the study revealed that respondents who owned livestock had higher knowledge compared to those who did not. Similar results have been reported where livestock owners have a higher knowledge as compared with non‐livestock owners (Abdela et al. 2017; Hagos et al. 2020). This observation has been supported by similar studies, where livestock owners demonstrated better knowledge of animal health, compared to non‐livestock owners (Abdela et al. 2017; Hagos et al. 2020). The direct exposure to animals and the challenges of livestock management likely increase their awareness of common diseases and their prevention methods.

The study also found that respondents with higher education levels (college and above) were more likely to adopt better preventive practices for managing livestock diseases. This aligns with previous findings from Bahir Dar, Ethiopia, where respondents with higher educational levels exhibited more proactive health practices, compared to those who had not attended formal schooling (Guadu et al. 2014; Abunna et al. 2024; Taye 2021). This is not surprising, as educated individuals often have better access to information and are more likely to act on knowledge about disease prevention. Farmers with higher education levels tend to be more progressive in adopting modern livestock management and disease prevention practices. In addition, the use of participatory epidemiology (PE) has been shown to be an effective approach for the early detection and monitoring of livestock diseases. PE actively engages farmers in surveillance and reporting, allowing for timely identification of emerging diseases and the integration of local knowledge into broader veterinary health strategies (Kholik et al. 2024a, 2024b). This could be a timely reminder for public health education service providers to make their prior focus on individuals who did not attend formal school learning. This highlights a critical gap in health and education services, suggesting that public health education efforts should focus more on individuals with lower educational backgrounds. Such initiatives could play a pivotal role in improving livestock health management and reducing the impact of zoonotic diseases. By targeting those who have not received formal schooling, health education campaigns can bridge the knowledge gap and promote better practices in disease prevention (Sadati et al. 2010).

## 6 Conclusion and Recommendation

This study revealed that while farmers in the East Gojjam Zone, Amhara Region, Ethiopia, demonstrate good knowledge (57%) and positive attitudes (69%) towards livestock disease management, their preventive practices are notably low (49%). The study highlights several critical challenges in livestock disease management in rural Ethiopia, especially in the East Gojjam Zone. Key issues include limited veterinary services, inadequate use of PPE, reliance on traditional treatments, irregular deworming practices and unsafe disposal of animal carcasses. To address these, there is a pressing need for improved veterinary infrastructure, better access to PPE and medications, enhanced education and awareness campaigns and public health interventions. Emphasising education and modern veterinary practices can significantly improve livestock health, reduce zoonotic disease risks and enhance rural livelihoods. This gap between knowledge and practice is largely due to inadequate veterinary service delivery, limited access to resources and structural barriers such as poor education and lack of infrastructure. Despite favourable attitudes towards livestock disease control, the limited implementation of best practices underscores the need for enhanced veterinary support, education and community involvement in disease prevention. In this context, the adoption of PE methods, such as participatory rural appraisal (PRA), can play a vital role in Ethiopia by actively engaging farmers in disease surveillance, early detection and reporting, thereby improving the effectiveness of livestock health management and bridging the knowledge–practice gap.

## Author Contributions

LY designed the study, performed the study, analysed and interpreted the data and wrote the paper. HY, KW, AT, DH conceived and designed the study, analysed and interpreted the data and wrote the paper. HY, NT and conceived and designed the study, drafting the article. YS conceived and designed the study and acquired data. All the authors participated in the final approval of the version to be submitted.

## Funding

This study was supported by Debre Markos University, College of Agricultural and Natural Resource.

## Ethics Statement

The study was approved by the Ethical Clearance Committee of the Institutional Research Ethics Review Committee (DMU, Ethical clearance Committee ref. no. 876/10/2024), Debre Markos University, Ethiopia. Verbal informed consent was approved by Debre Markos University Institutional Research Ethics Review Committee. For illiterate participants, the consent was provided through their legally authorized representatives based on the surrounding community rule and regulation (*kebele* and village leaders), who have legal responsibility and right in such kind of issue as usual. Consent form for participation and ethical consideration found in the Supporting Information as Appendix 2. All the methods were carried out in accordance with relevant guidelines and regulations.

## Conflicts of Interest

The authors declare no conflicts of interest.

## Supporting information




**Supporting File 1**: vms370743‐sup‐0001‐SuppMat.docx

## Data Availability

The data that support the findings of this study are available on request from the corresponding author. The data are not publicly available due to privacy or ethical restrictions.
