# Supplementary material for: Knowledge, Attitudes and Practices Regarding Livestock Diseases Among Residents of East Gojjam Zone, Amhara Region, Ethiopia
Source: Vet Med Sci. 2025 Dec 28;12(1):e70743. doi: 10.1002/vms3.70743 (PMC12745495; doi:10.1002/vms3.70743)
Supplement: Supplementary file 1 — Supporting File 1: vms370743‐sup‐0001‐SuppMat.docx [file VMS3-12-e70743-s001.docx]

**Appendix 1. Questionnaire Format for Questioner Survey for Livestock Disease Profile**

**Researcher name: __________________________District, _____________Kebele__________**

**Part A: Respondent Sociodemographic information**

1. Name of the respondent _________________________________
2. Sex ________
3. Age _________
4. Educational status:

A. Illiterate B. Primary school (1-8) C. Secondary school (9-12) D. College and University

1. Religion: ( Tick √ sign): Orthodox ______, Muslims _____, Protestant, ______Others_________
2. Marital status: ( tick √ sign): Single _____ Married _________Divorced____ Widowed____
3. Income status per month :______________ Ethiopian birr
4. What are your livelihood practices? **Rank** it

Crop production ___, Livestock production ___, Mixed production, ___ Trading____, Others___.

1. Have you livestock? A/ Yes B/ No
2. What type of livestock farming system you practiced?

A/ Extensive B/ Semi- intensive C/ Intensive

**Part B: Livestock Demography and The Management System**

1. Which age category of **animal** is dominant?

A, Young B/ Adult C/ Old

2. What is your **main** purpose of keeping the livestock?

A. Consumption (egg, milk, meet for special holiday), B. Cash income C. Drought power D. Transportation E. Dowry and gift F. Other specify_____

3. Who takes care of the livestock? A/ Father B/ Mother C/ Children D/ All

4. From where your animal get water and feed? A/ Communal B/ Private water source C/ others, specify___________________________

5 Housing: **A)** Fenced stable _________ **B)** House barn___________ C) Others _____

6. How did you get the animals? A/ Gift B/ Purchase C/ Restocking programs E/ Other (Specify) ___

**Part C: Questionnaire for the assessment of knowledge related with Livestock disease**

1. Do you know Livestock disease? (Tick √ choice): Yes _____ (1/1) No ______ (0/1)

2. Mention at least three **constraints** in livestock production in your area? Count the number of correct answers

| - Feed shortage |
| --- |
| - Disease |
| - Low genetic potential |
| - In adequate management |
| - Poor reproductive performance |
| **Result_____/5** |

3. Mention at least five **common diseases in your** livestock? Count the number of correct answers

| **Cattle D^ts^ name** |  |  |
| --- | --- | --- |
| - Anthrax | - Gastro intestinal parasitism |  |
| - Lumpy skin disease | - Fachiolosis |  |
| - Bloat | - Pneumonia/ pasteruolosis |  |
| - CBPP | - Skin disease /dermatitis |  |
| - Foot and mouth disease | - Mastitis |  |
| - Tuberculosis | - Brucellosis |  |
| - Black leg | - Trypanosomiasis |  |
| - Abortion |  |  |
| **Result_____/15** |  |  |
| **Sheep and Goat D^ts^ name** |  |  |
| - Shoat pox | - Gastro intestinal parasitism | |
| - Pasteruolosis/ pneumonia | - Anthrax | |
| - Orf (Mouth sore) | - Skin disease /dermatitis | |
| - Pest des petiis ruminants (PPR) | - Coenuruses/ Circling disease | |
| - Cowdrosis | - Contagious caprine pleuro pneumonia (CCPP) | |
| **Result_____/10** |  | |

4. Could you **identify** diseased animal from healthy? (Tick √ choice) Yes _____ (1/1) No ______ (0/1)

5. Mention at least **four** clinical sign of diseases animal from healthy

| - Loss of appetite | - Diarrhea |
| --- | --- |
| - Weakness | - Unthriftness |
| - Anemia | - Poor body condition |
| - Cough | - Sudden Death |
| - Rough hair coat |  |
| **Result_____/9** |  |

6. Do you know the disease can be transmitted from animal to human? (Tick √ choice)

| Yes ____ (1/1) | no____ (0/1) |
| --- | --- |

7. Do you know the disease can be transmitted from human to animal?

| Yes ____ (1/1) | no____ (0/1) |
| --- | --- |

8. Mention main **sources** of disease/infection transmission?

| - Ingestion | - Sexual transmission |
| --- | --- |
| - Air born | Inoculation and hospital (during treatment and vaccination |
| - Contact | **Result_____/5** |

9. Do you know animal disease can be obtained from Market?

| Yes ____ (1/1) | no____ (0/1) |
| --- | --- |

10. Do you know the difference susceptibility of Livestock disease across different age, breed and sex of animals?

| Yes ____ (1/1) | no____ (0/1) |
| --- | --- |

11. Do you know any medical disease control methods for disease animal? (Tick √ choice)

Yes _____ (1/1) No ______ (0/1)

12. Methods of livestock disease prevention and control approach

| - Quarantine | - Vaccination | |
| --- | --- | --- |
| - Isolation/ Movement restriction | - Stamping out |  |
| - Prophylaxis | - **Result_____/5** |  |

13. Do you know the availability of vaccines and animal drug for the prevention of livestock disease? (Tick √ choice)

Yes _____ (1/1) No _____ (0/1)

14. Is all livestock disease can be a preventable by vaccination only? (Tick √choice):

Yes _______ (0/1) No ___ (1/1)

15. Do you know the interval of vaccination for animal? (Tick √ choice):

Yes ____ (1/1) no____ (0/1)

**Part D: Questionnaire for measuring attitudes of the participants** (encircle)

1. Is livestock disease is economic and zoonotic important?

**a)** Strongly agree **b)** Agree **c)** Not sure **d)** Disagree **e)** Strongly disagree

2. Is spread of livestock disease can be prevented?

**a)** Strongly agree **b)** Agree **c)** Not sure **d)** Disagree **e)** Strongly disagree

3. Do you believe vaccination and prophylaxis of animals can prevent against disease?

**a)** Strongly agree **b)** Agree **c)** Not sure **d)** Disagree **e)** Strongly disagree

4. Livestock vaccine and drug is affordable.

**a)** Strongly agree **b)** Agree **c)** Not sure **d)** Disagree **e)** Strongly disagree

5. How is your willingness to vaccinate and prophylaxis your animals?

a) Not willing **b)** Not really willing **c)** Undecided **d)** Somewhat willing **e)** Willing

6. How satisfied are you with the vaccination campaign provided for the community animals?

a) Extremely dissatisfied **b)** Dissatisfied **c)** Neither dissatisfied nor satisfied

**d)** Satisfied **e)** Extremely satisfied [

7. Do you think cost of vaccination is factor for not using vaccination?

**a)** Strongly agree **b)** Agree **c)** Not sure **d)** Disagree **e)** Strongly disagree

8. Will you report sick or dead animals to the local authorities/ veterinary officers?

**a)** Strongly agree **b)** Agree **c)** Not sure **d)** Disagree **e)** Strongly disagree

9. Health care providers can handle disease outbreaks very well?

**a)** Strongly agree **b)** Agree **c)** Not sure **d)** Disagree **e)** Strongly disagree

10. Traditional healers can effectively treat infection against disease.

**a)** Strongly agree **b)** Agree **c)** Not sure **d)** Disagree **e)** Strongly disagree

11. How is your willingness to search information on disease outbreak?

**a)** Strongly agree **b)** Agree **c)** Not sure **d)** Disagree **e)** Strongly disagree

**Part E:** Questionnaire for measuring the practices of the participants

1. Separating diseased animal from health one? (Tick **√** choice): Yes ___ (1/1), No ___ (0/1),

2. Avoid slaughtering sick animals for meat consumption? (Tick **√** choice): Yes ___ (1/1), No ___ (0/1)

3. Avoid drinking raw milk from sick animal ? (Tick **√** choice): Yes ___ (1/1), No ___ (0/1)

4. Satisfactory veterinary service in your area? (Tick **√** choice): Yes ___ (1/1), No ___ (0/1)

5. Treating your animals when sick? (Tick **√** choice): Yes ___ (1/1), No ___ (0/1)

6. Do you use personal protective equipment when dealing with sick animals

Yes ___ (1/1), No ___ (0/1

7. Which type of medical advance you seek,

Traditional _____ (0/1)

Modern _____ (1/1)

8. Use botanical remedy for treating your sick livestock? (Tick **√** choice):

Yes __ (1/1), No ___ (0/1)

9. Vaccinating your animals against the disease? Tick **√** choice): Yes __ (1/1), No ___ (0/1)

10. Deworming and prophylaxis for animals? Tick **√** choice): Yes __ (1/1), No ___ (0/1)

11. Reduce disease occurrence by avoiding contact with neighboring animals? Tick **√** choice):

Yes __ (1/1), No ___ (0/1)

12. Way of dispose your died animals

A/ Throw on street b/ Burial c/ Burning

**Appendix 2: Consent Form**

Questionnaire for Knowledge, Attitudes, and Practices Regarding Livestock Diseases Among Residents of East Gojjam Zone, Amhara Region, Ethiopia

I am ………... From Debre Markos University who is doing research Knowledge, Attitudes, and Practices Regarding Livestock Diseases Among Residents of East Gojjam Zone, Amhara Region, Ethiopia. Your contribution for achieving the objective of the research is highly valuable, and incase if you don’t want to participate in the interview process you are not obligated to finalize the interview.

I have read the above information sheet or it has been read to me. The study has been explained to me in detail, and I have had a chance to ask questions and received satisfactory answers. I understand that if I have any other questions later about the research or about my rights as a research subject, I can call the Ethical Review Committee. I also understand that I will receive a copy of this Consent Form if I choose to participate.

Please select one:

 _____ I agree to participate in this project.

 _____ I do not wish to participate in this project.

Name of participant: _________________________

Date: _________________

Signature of participant: _____________________________

(Or thumbprint if the individual cannot write)

WITNESS (Only necessary if the individual cannot read/write):

Name of Witness: ____________________________________

Signature of Witness: _________________________________

Date: _______________

Thank you in advance for your cooperation!!

**Enumerator Name ____________________Signature __________Date ___________**
